# Reflections on HLA Epitope-Based Matching for Transplantation

**DOI:** 10.3389/fimmu.2016.00469

**Published:** 2016-11-28

**Authors:** Rene J. Duquesnoy

**Affiliations:** ^1^University of Pittsburgh Medical Center, Pittsburgh, PA, USA

**Keywords:** HLA, epitope, eplet, antibody, mismatch acceptability

## Abstract

HLA antibodies are primary causes of transplant rejection; they recognize epitopes that can be structurally defined by eplets. There are many reviews about HLA epitope-based matching in transplantation. This article describes some personal reflections about epitopes including a historical perspective of HLA typing at the antigen and allele levels, the repertoires of antibody-verified HLA epitopes, the use of HLAMatchmaker in determining the specificities of antibodies tested in different assays, and, finally, possible strategies to control HLA antibody responses.

## Introduction

It is now universally accepted that HLA matching affects transplant outcome and that HLA antibodies are primary causes of transplant rejection. HLA antibodies recognize epitopes, which can be structurally defined by eplets, i.e., small configurations of polymorphic amino acid configurations on the HLA molecular surface. This article addresses the concept that HLA matching can be determined at the epitope level. It is not intended as an extensive review of the literature; the www.HLAMatchmaker.net website has numerous epitope-related publications, and there are several review articles (1–4). Rather, this paper offers some recent reflections about the role of HLA epitopes in histocompatibility.

## Epitopes and a Historical Perspective of Serological HLA Typing

HLA emerged from observations by a few investigators including Rose Payne, Jon van Rood, and Jean Dausset who during the early 1960s studied sera with leukocyte antibodies in patients with non-hemolytic transfusion reactions and in women after pregnancies (5). Most reactivity patterns with leukocyte panels were uninterpretable, until international HLA workshops were organized whereby collaborating laboratories adapted the so-called microdrop complement-dependent lymphocytotoxicity technique developed by Terasaki and McClelland (6). Sera could be grouped into non-overlapping clusters with highly correlated reactivity patterns, and this permitted assignments of specificities such as HLA-A1, A2, B5, and B7. Such clusters served as reference standards for serological HLA typing reagents. Later on, subclusters of sera identified the so-called splits such as A10 was split into A25 and A26, and B16 was split into B38 and B39. Continued workshop efforts led to a set of HLA-class I specificities also called antigens that could be identified serologically with the complement-dependent lymphocytotoxicity technique. Most HLA antigens were defined with the so-called monospecific sera, but many others could be only identified from reactivity patterns of selected sera on typing trays.

Yunis and Amos first proposed the HLA-D locus from cellular assays based on mixed lymphocyte reactivity (7). Specific Dw determinants were later identified with HLA-D homozygous typing cells and primed alloreactive lymphocytes. Certain sera had antibodies with blocking effects on lymphocyte reactivity and with complement-dependent cytotoxicity assays using B-cells, and it was possible to identify serum clusters specific for serologically defined Dw related or DR antigens now referred to as DR1, DR2, DR3, etc. (8). Subclusters of sera also identified “splits” such as DR11 and DR12 of DR5.

HLA workshop studies during the 1980s identified clusters of sera specific for the DRB3/4/5-encoded antigens DR51, DR52, and DR53 and the DQB antigens DQ1, DQ2, DQ3, and DQ4 and again subclusters of sera demonstrated “splits” such as the DQ5 and DQ6 splits of DQ1.

As noted above, serological typing was primarily based on reactivities of specific antisera with antigens. Since it is now recognized that HLA antibodies are specific for epitopes rather than antigens, it seems obvious that HLA-typing sera must recognize distinct epitopes uniquely present on serologically defined antigens. The HLAMatchmaker analysis has shown that many HLA antigens detected by the so-called monospecific or duospecific sera have unique eplets, and almost all of them are recorded in the International HLA Epitope Registry (http://www.epregistry.com.br) as experimentally verified with informative antibodies (Table 1). In other words, anti-A1 antibodies actually recognize the 163RG eplet, which is only found on A1, anti-B7 antibodies are specific for 177DK uniquely present on B7, and anti-Cw1 antibodies recognize an epitope defined by 6K. Several serological splits can be explained by eplets paired with other residue configurations. For instance, A10 corresponds to 149TAH, whereas the A25 and A26 splits represent 149TAH + 80I and 149TAH + 80N, respectively. Similarly, the B38 and B39 splits of B16 are defined by antibodies specific for epitopes defined by the 158T + 80I and 158T + 80N pairs, respectively. Also, Table 1 illustrates that most serologically defined DR and DQ antigens have uniquely distinct eplets. The cellularly defined DP specificities [originally called SB (9)] do not have unique eplets, and this explains why they cannot be readily determined serologically with monospecific sera.

**Table 1:** **Specificities of commonly used serological typing reagents and their corresponding eplets**.

Serological specificity	Corresponding eplet	Serological specificity	Corresponding eplet	Serological specificity	Corresponding eplet
Al	163RG	B18	30G	DR7 + 9	78V_2_
A1 + A36	44KM_2_	B18 + B35	44RT	DR8	25YRF
A2	66RKH	B27	71KA	DR8 + 12	16Y
A2 + A28	142MT	B40	44RK	DR9	13FEY
A2 + A69	107W	B44	199V	DR10	40YD_2_
A3	161D	B48	245TA	DR11	57DE
A9	66GKH	Bw4	82LR	DR12	37L
A10	149TAH	Bw6	80ERN	DR13	71DEA
A11	151AHA	Cwl	6K	DR14	57AA
A25 + A32	76ESI	Cw2	211T	DR15	71A
A29	62LQ	Cw3	173K	DR17	26TYD
A30	152RW	Cw4	17WR	DR51	96EN_3_
A30 + A31	56R	Cw5 + 8	138K	DR52	98Q
A31 + A33	73ID	Cw7	193PL	DR53	48YQ_6_
A68	245VA	DR1	12LKF_2_	DQ1	52PQ_2_
B5 + B35	193PV	DR1 + 10	13FEL	DQ2	45GE_3_
B7	177DK	DR1 + 51	96EV	DQ3	55PP
B8 + Cw7	9D	DR2	142M_2_	DQ4	56L_2_
B12	167ES	DR3	74R	DQ5	74SR_3_
B13	144QL	DR4	96Y_2_	DQ6	125G
B15	163LW	DR3 + 6	31YYFH	DQ7	45EV
B16	158T	DR7	25Q_3_	DQ8	56PPA
B17	71SA			DQ7 + 9	56PPD

*Eplet descriptions are according to HLAMatchmaker (www.epitopes.net). The number represents the sequence location of one of the polymorphic residues annotated with standard single letters. Some eplets have subscripted numbers indicating additional residue configurations in other locations*.

The information in Table 1 is based on molecular structure and amino acid sequence information of HLA antigens which did not emerge until after the late 1980s. Before that, the specificities of serum clusters in the early workshops could only be designated with an arbitrary notation system of serologically defined antigens, although we can now readily see that they reflect the recognition of distinct epitopes. Accordingly, the HLA antigen-matching effect on transplant outcome can be reinterpreted as actually demonstrating the influence of matching for epitopes, albeit limited numbers were considered in these association analyses.

## Epitopes and Serologically Cross-Reacting Groups

Many sera with HLA antibodies exhibit complex reactivity patterns that prohibit their use in serological typing. Early studies identified sera that reacted with the so-called cross-reacting groups (CREGs) of HLA antigens; the A2-CREG and B7-CREG are common examples (10, 11). Each CREG has the so-called public determinants shared between certain groups of antigens and so-called private determinants limited to a given serologically defined antigen within the CREG (12). Many public determinants correspond to structurally defined epitopes. As an example, the A2-CREG, which includes A2, A23, A24, A68, A69, and B17, has several epitopes corresponding to public determinants including the 127K eplet on A2 + A23 + A24 + A68 + A69, 144TKH on A2 + A68 + A69, 107W on A2 + A69, and 62GE on A2 + B17, and all of them have been antibody verified. It has become evident that all HLA class I antigens have public determinants or shared epitopes that can be recognized by antibodies.

Although CREG matching has been applied to the identification of compatible platelet donors for refractory alloimmunized thrombocytopenic patients (13), no beneficial effect could be convincingly demonstrated on transplant outcome largely because these studies did not consider individual public epitopes. Most highly sensitized patients have antibodies against public epitopes that can now be defined structurally.

## Epitope-Based Matching Requires DNA-Based HLA Typing at the Allele Level

Serological HLA typing had always accuracy problems because of the lack of reagents and technical limitations. After elucidation of the HLA molecular structure and nucleic acid sequencing of HLA antigens during the late 1980s, the application of DNA-based technologies permitted accurate HLA antigen typing results. Very soon, many antigens were identified with amino acid differences, and this led to assignments of alleles to be annotated with a colon (:) followed by two and later on three digits after more than 100 alleles corresponding to the 2-digit antigen had been identified (Examples are A*02:01 and A*02:101). Certain amino acid differences affect the expression of eplets. For instance, A*24:02 has the antibody-verified 166DG eplet shared with A*01:01, A*23:01, A*80:01, and B*15:12, whereas A*24:03 has 166EW shared with A*02:01, A*03:01, etc. This example illustrates the difficulty of matching at the antigen level, in this case A24 if the patient has antibodies against 166DG or 166EW. It is now recognized that HLA compatibility is better determined at the allele than at the antigen level (14).

There are thousands of HLA alleles and the question arises which ones should be typed for in the clinical transplant setting. One might focus on alleles present in the patient and donor population of a given transplant program. Although rare alleles might be excluded it is now apparent that because of the increasing racial and ethnic heterogeneity of most populations such alleles occur more frequently as mismatches. Tissue typing techniques are moving forward very fast in the clinical setting, and each HLA allele has precise information about its eplet repertoire. The degree of eplet mismatching of a donor allele depends on the HLA phenotype of the recipient, which has its own repertoire of self-eplets. Such determinations can be readily made with specifically designed computer programs such as HLAMatchmaker.

## Antibody Reactivity with HLA Epitopes

Epitope-based HLA compatibility determination requires a basic understanding of how antibodies interact with epitopes. Antibody reactivity testing with HLA panels can be done with different techniques from immunoglobulin and complement component C1q binding to isolated HLA molecules attached to microbeads to flow cytometric binding on lymphocytes and complement-dependent lymphocytotoxicity. These methods may give different results which make clinical relevance assessments difficult. Epitope specificity analyses of antibodies may clarify some of these issues.

**Figure 1:**
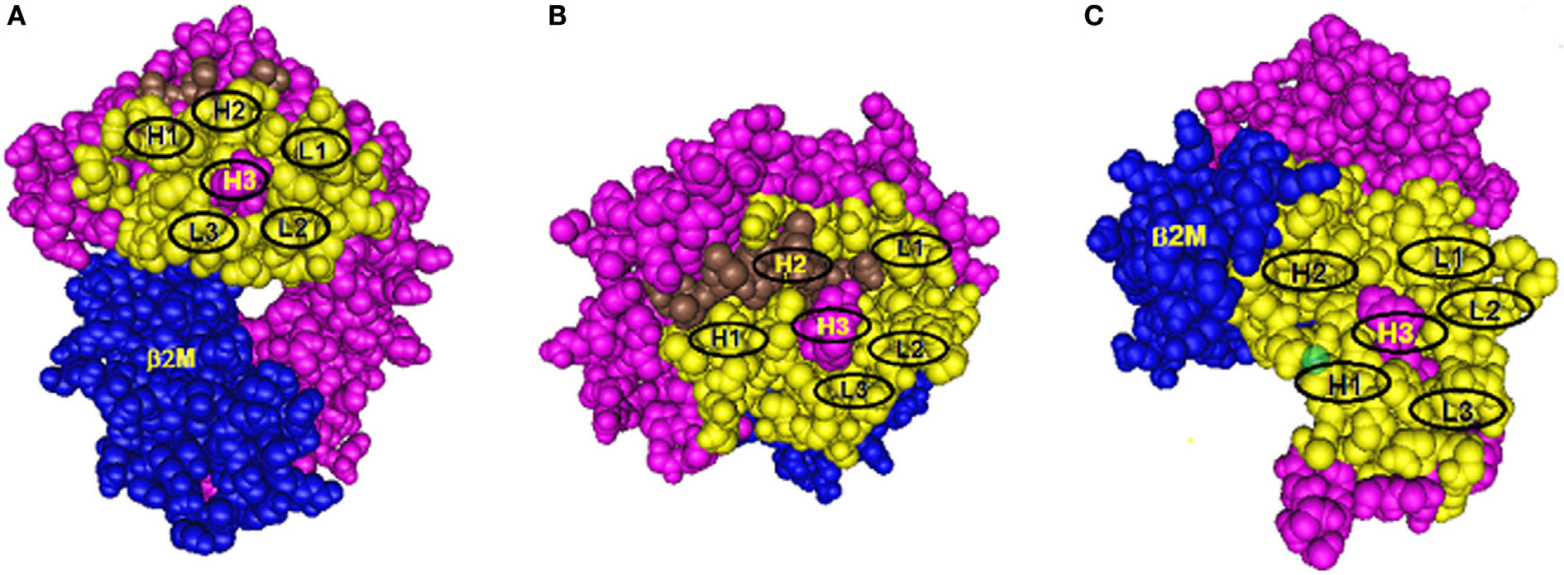
**Three models of structural HLA class I epitope**. The HLA molecule has three components: HLA chain (pink), β2-microglobulin (blue), and the bound peptide (green). The centrally located eplet (in pink) interacts with CDR-H3. Residues within a 15-Å radius are colored yellow and include configurations (in oval circles) that make contact with other CDRs on heavy chain (H1 and H2) and light chain (L1, L2, and L3). **(A–C)** reflect three different epitope locations on the molecule.

Antibody binding to protein epitopes occurs through six complementarity-determining region (CDR) loops, three of them H1, H2, and H3 are on the immunoglobulin heavy chain and L1, L2, and L3 are on the light chain. Each loop interacts with a small set of amino acid residues in the so-called structural epitope and CDR-H3 which binds to the so-called functional epitope (or hot spot) in a central location has a dominant role in determining antibody specificity. Eplets are considered to be equivalent to functional epitopes. Figure 1 shows models of eplets in three different locations on class I molecules and in context with corresponding structural epitopes and hypothetical contact sites for the CDRs of antibody. Two eplets are readily antibody accessible on the upper domains (Figures 1A,B), but antibodies to eplets on the membrane-proximal domain (Figure 1C) might interact with only solubilized but not with lymphocyte membrane-bound HLA molecules because these epitopes may not be readily antibody accessible. Would such epitopes be clinically significant?

Antibodies through their CDR-H3 recognize specific eplets centrally located in the structural epitope. Other eplets in the same sequence location but with different residue compositions are generally non-reactive, but there can be exceptions if the eplet has a high degree of residue homology with the eplet that had induced the antibody. As an example, the 82LR eplet, which describes the well-known Bw4 epitope, has two closely nearby residues 80I and 80T. Many 80I + 82LR-induced antibodies recognize also 80T + 82LR and binding strengths can vary from high to low. This is an example of the so-called Landsteiner type of serological cross-reactivity, whereby different but structurally related epitopes react with the same antibody. It should be noted that certain 80I + 82LR-induced antibodies never react with 80T + 82LR; they can be designated as specific for only 80I. This epitope-based analysis seems helpful in the assessment of the mismatch acceptability of 82LR (or Bw4) when 80T + 82LR-carrying alleles have no or very low reactivity with patient’s serum.

As illustrated in Figure 1, the structural epitope has other amino acid configurations that interact with the remaining CDRs of antibody. As described in many immunology textbooks, an important consideration is the so-called affinity maturation process during the antibody response, whereby DNA regions corresponding to CDRs undergo mutations which increase antibody affinity with the structural epitope. As an example, let us just consider for Figure 1A one CDR-loop L2, which as a result of affinity maturation, has increased binding with a given amino acid configuration in the structural epitope of the immunizing allele. Two possible configurations can be considered. One comprises only monomorphic residues shared between all alleles in the panel, and the epitope recognized by antibody corresponds solely to an eplet. Second, this configuration has polymorphic residues and only alleles that share such residues with the immunizing allele are antibody reactive. Such residues represent critical contact sites for antibody. Accordingly, these epitopes can be defined by eplets paired with distinct residue configurations (including eplets) in other sequence locations within a 15-Å radius, a presumed dimension of a structural epitope. As an example, there are antibodies specific for 82LR paired with 145RA; this epitope is present on all 82LR-carrying alleles except A25 and B13 that have 82LR + 145RT and 82LR + 145LA, respectively. It should be noted that the critical contact residue configurations are almost uniformly present on at least one allele of the antibody producer, and this suggests an autoimmune component of the antibody response to a mismatched eplet.

As illustrated in Figure 1B, some CDRs can make contact with peptides bound to the groove, and it is possible that peptides serve as critical contact sites for a CDR such as H2. Indeed, it has been reported that certain HLA antibodies are peptide dependent (15–17).

For many protein antigen–antibody complexes, there is a certain level of permissiveness for residue substitutions in critical contact areas, and this applies also for HLA epitopes. Certain residue substitutions in the structural epitope have a moderate effect on an allele’s reactivity with antibody, whereas others are inhibitory to the level of weak reactivity or non-reactivity of specific eplet-carrying alleles. The latter might be considered as epitope-based acceptable mismatches.

The same rules apply to epitope descriptions of class II alleles which more often solely correspond to eplets. Only few DRB epitopes correspond to eplet pairs, and this might be due to monomorphic residue nature of the DRA chain. Several immunogenic DRB1 eplets are also on alleles encoded by DRB3, DRB4, and/or DRB5. Each of them has distinct antibody-verified eplets, and it seems that epitope matching for DRB3/4/5 affects the class II antibody response.

DQ and DP encode for heterodimers of A and B chains which are both polymorphic and have distinct eplets many of which have been antibody verified. DQ-specific antibodies appear most prevalent among antibodies induced by class II mismatches. Such antibodies are specific for eplets on DQA and DQB chains, and there is emerging evidence that some DQ epitopes are defined by eplet pairs involving both chains (18, 19). This suggests that epitope-based matching should consider the DQ heterodimer rather than the individual chains alone. DP mismatching involves generally fewer epitopes on DPB and especially on DPA; immunogenic eplets, such as 84DEAV and 55DE, are present on large groups of DPB alleles.

## Repertoires of Antibody-Verified HLA Epitopes

Eplets are small configurations of amino acid residues that play dominant roles in HLA epitopes reactive with antibodies. Such configurations are theoretical considerations based on residue differences in polymorphic sequence locations, but we must raise the question how many of them are actually recognized by specific antibodies. One would expect the clinical relevance of epitope-based matching to apply only to epitopes that have been experimentally verified with informative antibodies. The HLA Epitope Registry has a list of antibody-verified epitopes recorded this far for each locus, but the repertoire is still incomplete. Very recently, the website includes a downloadable PDF file “EpiPedia of HLA” that describes the antibody verifications of HLA epitopes in detail. With the help of participating HLA laboratories that might have interesting serum antibody reactivity patterns, we will continue our analyses to identify new epitopes. The HLAMatchmaker website www.epitopes.net (formerly www.HLAMatchmaker.net) has now a downloadable Excel document “Five Maps of HLA Epitopia,” which describes the sequence locations of antibody-verified eplets and polymorphic residues as potential candidates defining additional epitopes. These maps can be used in navigating the continents of HLA Epitopia while searching for newly antibody-defined epitopes (20).

## Technique-Dependent Epitope Specificities of HLA Antibodies

The use of C1q-binding and complement-dependent lymphocytotoxicity assays has added other dimensions of the complexity of the antibody response to a mismatch. These tests are based on sequential events following the formation of the antibody–epitope complex. C1q binding requires a conformational change in the antibody molecule, thereby exposing the C1q receptor on the Fc part, and complement-dependent cytotoxicity is initiated by the activation of antibody-bound C1qrs complex as the first step of the classical complement pathway. Both processes require free energy released during antibody–epitope complex formation, and the amount depends on the binding strength between all CDRs with the structural epitope. Some antibodies react only with the immunizing epitope in Ig-binding assays; this means that the amount of free energy release is insufficient for the activation of complement-dependent mechanisms that contribute to inflammatory responses (21, 22). A given antibody can react with the immunizing eplet and certain eplet-sharing alleles in the panel in all three assays. Other eplet-carrying alleles react only in Ig-binding assays, because they lack certain critical residues in the corresponding structural epitopes required for strong binding with antibody required for the initiation of the inflammatory process. Are such alleles acceptable or unacceptable mismatches?

## Pretransplant Serum Testing for Epitope Specificities of HLA Antibodies

The above interpretations about antibody reactivity and epitope specificity are more readily made with monospecific sera. However, most sera from sensitized patients have mixtures of antibodies, and although the reactivity patterns are generally limited to a few specificities, there are additional features that can make epitope-based interpretations often quite challenging. They include unexpected reactivities of certain panel alleles due to “natural” antibodies or non-specific blocking factors including a prozone effect; sera may also have competing antibodies with different characteristics including Ig subtypes. Many sera from highly sensitized patients have antibodies reacting with high-frequency (i.e., >80%) epitopes, which make detections of antibodies against lower frequency epitopes more difficult unless these antibodies are separated through absorption–elution studies with selected alleles.

Technique dependencies of serum reactivity may also affect the interpretation of epitope specificities, especially for highly sensitized patients who have several antibody populations in different concentrations and affinities that affect their reactivity with HLA panels. Again, absorption–elution studies with selected alleles might dissect these serum reactivity patterns, so that an epitope analysis can be more readily done.

The HLAMatchmaker website has three downloadable antibody analysis programs in Excel format: HLA-ABC, HLA-DRDQDP, and MICA. The latest 02 versions focus on antibody-verified epitopes recorded so far in the HLA Epitope Registry. All of them correspond to single eplets or eplets paired with other residue configurations uniquely shared by a group of antibody-reactive alleles. The antibody analysis programs also include “other” theoretical eplets, which might become experimentally verified if informative antibodies are identified. The HLAMatchmaker website has a downloadable instruction manual for the epitope analysis of HLA antibodies tested in assays with single alleles.

The 02 versions require entering of the HLA information of the panel, the MFI values of each allele and the allelic HLA type of the patient and preferably the immunizer (e.g., a previous transplant or in case of a pregnancy, the paternal allele of a child). A calculation of the MFI for the self alleles offers a basis for determining a cutoff value be entered, and the program then determines which epitopes are shared between reactive alleles.

In the clinical setting, the primary purpose of the serum HLA antibody analysis of transplant candidates is to identify potential donors whose mismatched HLA alleles are acceptable. This approach is useful not only for organ transplantation but also for platelet transfusions of allosensitized thrombocytopenic patients. Eurotransplant has incorporated HLAMatchmaker in the Acceptable Mismatch Program to identify donors for highly sensitized patients (23, 24).

Epitope specificity analyses might also be useful in desensitization protocols to remove donor-specific antibodies (25). Such protocols are not always uniformly successful but for some patients they may remove some epitope-specific antibodies, thereby opening new windows of opportunity regarding the identification of selected allelic mismatches.

## Posttransplant Monitoring of HLA Antibodies

Many posttransplant studies have shown associations between the appearance of donor-specific antibodies with allograft rejection and failure. Most studies have cases whereby the transplant continues to function quite well in the presence of donor-specific antibodies, and this raises the question which antibodies are clinically significant in transplantation. By definition, such antibodies must be absorbed by the allograft where they recognize epitopes expressed on the vascular endothelium and other tissues and initiate inflammatory processes leading to rejection. Accordingly, testing for circulating HLA antibodies in the presence of a transplant has its limitations, and this becomes apparent with the increased serum reactivity often seen after allograft nephrectomy (26–28), and the identification of donor-specific antibodies in eluates from surgically removed transplants (29–33). The question about epitope specificities of clinically important antibodies can be studied in comparative analyses of pre- and post-allograft nephrectomy sera and better yet by analyzing antibody reactivity patterns of allograft eluates.

## Control of Antibody Responses to HLA Epitopes

HLA matching at the epitope level also benefits transplant outcome in non-sensitized patients who have no donor-specific HLA antibodies before transplantation. Numerous studies have demonstrated significant correlations between eplet loads of HLA mismatches and the development of donor-specific class I and class II antibodies as well as rejection incidence and allograft outcome (34–48). These findings are clinically useful in two ways. First, for each transplant, the eplet load can be readily determined with special matching programs that can be downloaded from the HLAMatchmaker website. The amount of a mismatched epitope load can be considered as a risk factor during the posttransplant monitoring for antibody-mediated rejection. This information seems also useful in clinical protocols to achieve immunological tolerance (25).

Second, eplet loads can be used to develop new donor selection strategies for non-sensitized recipients especially younger patients. Some transplant programs have already begun to implement this approach (49–51). HLA mismatches with low eplet loads can be expected to improve transplant outcome. Even if the first allograft rejected, retransplant candidates might become less highly sensitized, thereby making it easier to find acceptable mismatches.

The relative immunogenicity of HLA eplets plays an important role in mismatch permissibility of HLA alleles. This can be determined empirically by analyzing the frequencies of antibody responses to mismatched donor eplets which depend on the HLA phenotypes of the recipient and their specificities would be for epitopes defined by eplets or eplet pairs (52). Such studies do not consider the mechanisms of the antibody response which involves the activation of B-cells with epitope-specific Ig-receptors, their subsequent proliferation induced by cytokines from helper T-cells and the differentiation including affinity maturation into antibody-producing plasma cells.

Three recent concepts have begun to address these mechanisms. First, the non-self–self paradigm of HLA epitope immunogenicity is based on the hypothesis that HLA antibodies originate from B-cells with low-avidity, self-HLA epitope-specific Ig-type receptors that can interact with non-self eplets to generate a signal for B-cell activation. Such non-self eplets would be recognized by the specificity-determining CDR-H3, and in the context of the corresponding structural epitope they must be surrounded by self-residues that contact the other CDRs of antibody (53–56). Second, Kosmoliaptsis et al. have proposed that the relative antigenicity of an eplet can be predicted from the physiochemical properties of its amino acid residues (57–59). Accordingly, the electrostatic difference between a non-self eplet and a self-eplet might provide the trigger for B-cell activation (60). Third, activated B-cells need T-cell help for their proliferation and differentiation into antibody-producing plasma cells. The group of Eric Spierings has proposed the so-called PIRCHE-II concept (i.e., Predicted Indirectly ReCognizable HLA Epitopes presented by HLA-DRB1), whereby helper T-cells release cytokines upon recognition of DRB-presented mismatched HLA peptides generated after processing of Ig receptor–allele complexes taken up by activated B-cells (61, 62).

These are reasonable theoretical concepts, although no direct experimental methods are available to analyze the very early phases of the antibody response (63). At present, we can only use indirect approaches, such as serum antibody specificity analysis, molecular assessments of matching, and structural analysis of HLA-antigen–antibody complexes, to study the immunogenicity of HLA epitopes. It is important to know a complete repertoire of antibody-verified epitopes. Such investigations could lead to a better understanding of HLA epitope immunogenicity, and how this can be applied to permissible mismatch strategies.

## Conclusion

Epitope-based HLA matching has become a new concept in the clinical transplant setting. It relies on HLA typing at the allele level and can be used to identify acceptable mismatches for sensitized patients and to develop permissible mismatch strategies for non-sensitized patients. Our understanding of HLA epitopes is still in progress, and more studies are needed to identify antibody-verified epitopes to be recorded in the HLA Epitope Registry. Also, histocompatibility testing laboratories will have opportunities to sort out complex serum reactivity patterns and interpret technique-dependent epitope specificities of HLA antibodies and their clinical relevance. Sooner or later, there will be new epitope-based HLA-matching approaches with increased benefits to transplant patients.

## Author Contributions

The author confirms being the sole contributor of this work and approved it for publication.

## Conflict of Interest Statement

The author declares that the research was conducted in the absence of any commercial or financial relationships that could be construed as a potential conflict of interest.
